# Significance of location and extent of perineural invasion in early‐stage oral cavity squamous cell carcinoma

**DOI:** 10.1111/his.15406

**Published:** 2025-01-06

**Authors:** Shima Mohamed, Deirdre Callanan, Patrick Sheahan, Linda Feeley

**Affiliations:** ^1^ Department of Pathology Cork University Hospital Cork; ^2^ Department of Otolaryngology South Infirmary Victoria University Hospital Cork Ireland; ^3^ Department of Surgery University College Cork Ireland; ^4^ ENTO Research Unit College of Medicine and Health, University College Cork Cork Ireland

**Keywords:** head and neck cancer, oral cancer, perineural invasion, prognosis, squamous cell carcinoma

## Abstract

**Aims:**

Perineural invasion (PNI) is associated with survival in oral cavity squamous cell carcinoma (OCSCC). There is evidence to suggest that PNI location and extent may be of additional significance. The primary aim of this study was to evaluate the prognostic ability of PNI, including location and extent, in early‐stage OCSCC.

**Methods and results:**

This was a retrospective study, with the main cohort comprising of 129 patients with pT1/T2 pN0/Nx TNM8 OCSCC. Slides were re‐reviewed in cases reported as having PNI to classify location as intratumoural (IT) and/or extratumoural (ET) and extent as unifocal (UF) or multifocal (MF). Univariate and multivariate analysis assessing impact of pathological features on survival outcomes was performed. On multivariate analysis, IT PNI was significantly associated with locoregional recurrence‐free survival (LRS) [odds ratio = 5.69, 95% confidence interval (CI) = 1.50–21.63, *P* = 0.01]. Disease‐specific survival (DSS) and overall survival (OS) were non‐significant. In comparison, ET PNI was predictive of LRS (odds ratio = 20.57, 95% CI = 3.48–121.73, *P* = 0.001), DSS (odds ratio = 40.47, 95% CI = 5.17–316.96, *P* = 0.0004) and OS (odds ratio = 11.92, 95% CI = 2.18–65.22, *P* = 0.004). Multifocal PNI was significant on univariate analysis for all three outcome parameters evaluated, but these findings were not maintained on multivariate assessment.

**Conclusions:**

Extratumoural PNI is strongly predictive of survival outcomes, including OS, in early‐stage OCSCC. These findings support the reporting of PNI location as a mandatory data element. The impact of PNI extent requires further study.

## Graphical abstract

Extratumoural perineural invasion was strongly predictive of survival outcomes in our early‐stage oral cavity squamous cell carcinoma cohort. These findings support the reporting of location of perineural invasion as a mandatory pathological data element.

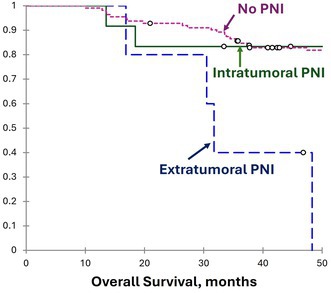

**Abbreviations**
DSS: disease‐specific survival
ET: extratumoural
IT: intratumoural
LRS: locoregional recurrence‐free survival
MF: multifocal
ND: neck dissection
OCSCC: oral cavity squamous cell carcinoma
OS: overall survival
PNI: perineural invasion
UF: unifocal

## Introduction

Perineural invasion (PNI) is a well‐established adverse prognosticator in oral cavity squamous cell carcinoma (OCSCC), and has been shown to be associated with nodal metastases and reduced disease‐specific and overall survival, including in early‐stage disease.^1^, ^2^, ^3^, ^4^, ^5^, ^6^, ^7^, ^8^, ^9^, ^10^ There is evidence to suggest that PNI that is extratumoural (ET) in location may be more significant.^11^, ^12^ The extent of PNI is another feature of potential importance.^12^, ^13^, ^14^, ^15^ However, neither PNI location nor extent are currently mandatory reporting parameters in the College of American Pathologist's (CAP) guidelines.^16^ In contrast, the updated Royal College of Pathologist's (RCPath) data set for oral cavity carcinoma states that PNI ahead of the invasive tumour front requires particular emphasis and is now included as a core data element.^17^

The primary aim of this study was to evaluate the prognostic ability of PNI in early‐stage OCSCC. The presence of any PNI, location of PNI [intratumoural (IT) versus ET] and extent of PNI (unifocal versus multifocal) were analysed with the specific objective of ascertaining whether PNI location or extent should be mandatory reporting elements. Secondary aims were to determine the impact of clinical parameters and other routinely reported histopathological features on patient outcomes. The study cohort comprised pathological T1/T2 cases staged using tumour, nodes, metastasis (TNM) classification, 8th edition (TNM8).

## Methods

This study was a retrospective review of patients who underwent primary surgical treatment for pT1/T2 (TNM8) OCSCC. Patients were treated surgically at the South Infirmary Victoria University Hospital, Cork, Ireland between the years 2007 and 2020. The Cork Clinical Research Ethics Committee granted ethical approval. Exclusion criteria were cases with tumour depth of invasion > 10 mm, patients with evidence of distant metastases at diagnosis, patients with prior or synchronous primary head and neck cancer and surviving patients with < 1 year of clinical follow‐up.

Cases and associated clinicopathological data were extracted from our cancer database, having been previously populated by review of medical charts, pathological reports and/or slide review. Clinical data recorded included sex, tumour subsite, smoking status, alcohol usage, adjuvant therapy and clinical outcome measures, including recurrence, death due to cancer and death due to other causes. Pathological data recorded included PNI, depth of invasion (DOI), pattern of invasion, lymphovascular space invasion, margin status and pathological stage (TNM8), including relevant nodal parameters required for staging. DOI measurements were previously re‐evaluated for all patients in the study cohort who underwent surgery prior to the 2017 publication of the updated AJCC definition for DOI.^18^ Margin positivity was categorised using the CAP definition of invasive carcinoma or high‐grade dysplasia present at margin.^16^ Margin status was based on the main specimen, excluding consideration of separately submitted frozen sections or extra tumour bed resections.

**Figure 1:**
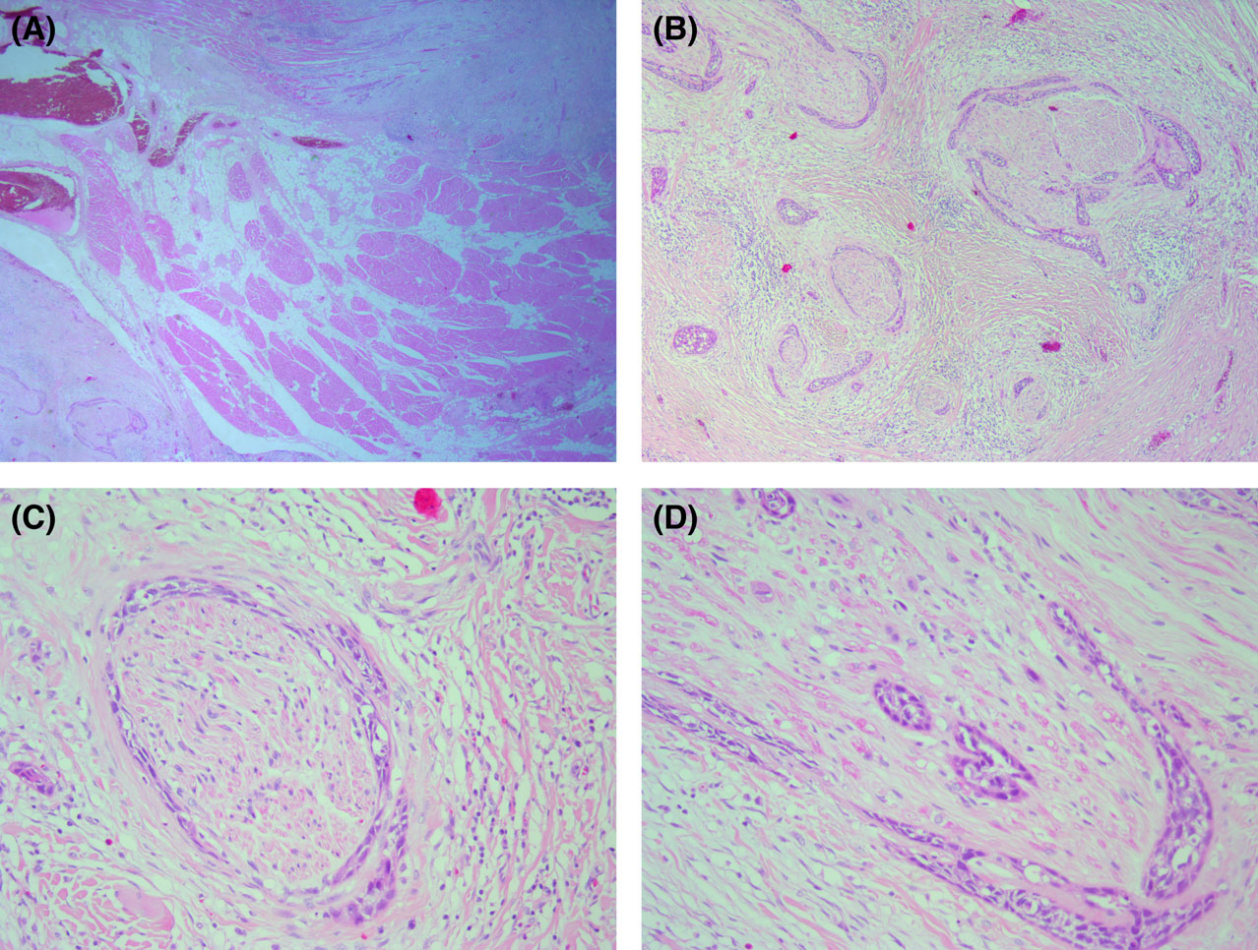
A, Extratumoural perineural invasion (ET PNI) bottom left, intervening muscle, invasive tumour front top right. **B**, Multifocal PNI. **C**, PNI characterised by near complete encirclement of the nerve by tumour. **D**. PNI characterised by partial encirclement of the nerve and invasion of the endoneurium.

Haematoxylin and eosin (H&E)‐stained slides of the primary tumour were re‐reviewed in cases reported as positive for PNI to classify the location as intratumoural and/or extratumoural, and to quantify extent as focal or multifocal. Slide review was performed by two pathologists at a multiheaded microscope and consensus opinion was recorded. The presence of PNI was reconfirmed by the two reviewing pathologists with a small number of cases recategorised from PNI‐positive to ‐negative, which was expected due to interobserver variability.^19^, ^20^ PNI was defined as tumour invasion of the nerve (perineurium, epineurium or intraneurium) and/or tumour wrapping around the nerve.^21^ ET PNI (versus IT PNI) was defined as PNI present ≥ 1 mm beyond the invasive tumour front. In our assessment of PNI extent/focality the potential for skip lesions, plane of section or involvement of branches of larger nerves was not taken into consideration. Rather multifocal PNI was simply defined as involvement of more than one nerve bundle, with this definition chosen to optimise reproducibility in everyday reporting. Nerve diameter was not analysed, given that PNI of nerves < 1 mm in diameter has been shown to be prognostic^2^ (Figure 1).

XLSTAT (Addinsoft, Paris, France, version 2015.1.03) was used for statistical analysis. Survival was measured from date of surgery to date of death or last clinical follow‐up. Patients dying with recurrence, uncontrolled cancer or from complications of treatment were classified as having died from disease.^22^, ^23^ Locoregional recurrence‐free survival (LRS) was measured from date of surgery to date of diagnosis of locoregional recurrence, or to last clinical follow‐up. Local recurrence was regarded to be present in those with recurrent cancer at the same or contiguous subsite as the original primary, regardless of interval since the initial surgery. New head and neck cancers not fulfilling the criteria for local recurrence were regarded to be true second primary head and neck cancers and were censored on the date of presentation with the second primary. Survival outcomes were calculated using the Kaplan–Meier method. Two separate analyses were performed. The first included only patients who either had pathologically negative necks (pN0) or clinically negative necks and did not undergo neck dissection (pNx). The second analysis additionally included those with cervical metastases (pN+).

Univariate and multivariate analysis assessing impact of clinicopathological features on survival outcomes was performed using Cox's proportional hazards modelling. For multivariate analysis, backward modelling was utilised and variables with *P*‐value < 0.1 on univariate analysis included. Proportionality of hazards was assessed by visual inspection of Kaplan–Meier curves. Goodness‐of‐fit was analysed using the χ^2^ test on the log ratio. A *P*‐value of 0.05 was deemed significant.

## Results

The entire study cohort comprised 159 T1/T2 TNM8 patients. A total of 106 patients underwent neck dissection (ND), which was negative for metastases in 76 (pN0) and positive in 30 (pN+). A further 53 patients with clinically and radiologically negative necks (cN0) who did not undergo ND were classified as pNx. For clinicopathological and demographic parameters of the full cohort see Table 1. Slide review was undertaken in the 32 cases (20.1%) reported as PNI‐positive. Two cases were reclassified on review from PNI‐positive to negative. Therefore, 30 patients (18.9%) were confirmed to be PNI‐positive, 21 of whom had multifocal PNI (13.2%) and five had extratumoural PNI (3.1%); see Table 2 for full breakdown of PNI subgroups.

**Table 1:** Clinicopathological and demographic features of study population, 159 T1/T2 TNM8 patients

		*n*	%
Sex	Male	97	61
	Female	62	39
Primary site	Tongue	76	47.8
	Floor of mouth	44	27.7
	Buccal	13	8.2
	Alveolus/RMT/palate	22	13.8
	Lip	4	2.5
Smoking status	Smoker	73	45.9
	Ex‐smoker	34	21.4
	Non‐smoker	38	23.9
	Unknown	14	8.8
Alcohol use	Yes	97	61
	Ex‐drinker	13	8.2
	No	33	20.7
	Unknown	16	10.1
Second head and neck primary	Yes	21	13.2
T‐classification (AJCC 8th edition)	T1	71	44.7
	T2	88	55.3
N‐classification	Nx	53	33.3
	N0	76	47.8
	N+	30	18.9
Extranodal extension	Yes	11	6.9
Depth of invasion	</= 5mm	84	52.8
	> 5mm, </=10 mm	75	47.2
Involved margins	Yes	14	8.8
Pattern of invasion	Non‐cohesive	58	36.5
Perineural invasion	Yes	30	18.9
Lymphovascular space invasion	Yes	10	6.3
Postoperative radiotherapy (RT)	No	109	68.5
	RT alone	44	27.7
	Chemoradiotherapy	6	3.8

RMT, retromolar trigone; AJCC, American Joint Committee on Cancer.

**Table 2:** PNI results, 159 T1/T2 TNM8 patients

PNI	Total *n* (%)	Unifocal PNI *n* (%)	Multifocal PNI *n* (%)
Any PNI	30 (18.9)	9 (5.7)	21 (13.2)
IT PNI only	25 (15.7)	8 (5.0)	17 (10.7)
ET PNI only	1 (0.6)	1 (0.6)	0
Both IT & ET PNI	4 (2.5)	0	4 (2.5)

IT, intratumoural; ET, extratumoural; PNI, perineural invasion.

Mean (median) follow‐up was 72 (65) months (range = 1–194 months). Mean (median) follow‐up for surviving patients was 93 (85) months (range = 13–194 months). Thirty‐six patients (22.6%) developed recurrence, including 25 patients with local recurrence (nine concurrent with regional recurrence), eight with isolated regional recurrence and nine with distant recurrence, including three with isolated distant disease. Forty‐four patients developed second primaries, including 21 in the head and neck, 11 of which were in the oral cavity or oropharynx; all were censored at the time of presentation with the second primary. Seventy‐nine patients (49.7%) died, 22 of whom (27.8%) died from the index OCSCC and 57 (72.2%) from other causes.

**Figure 2:**
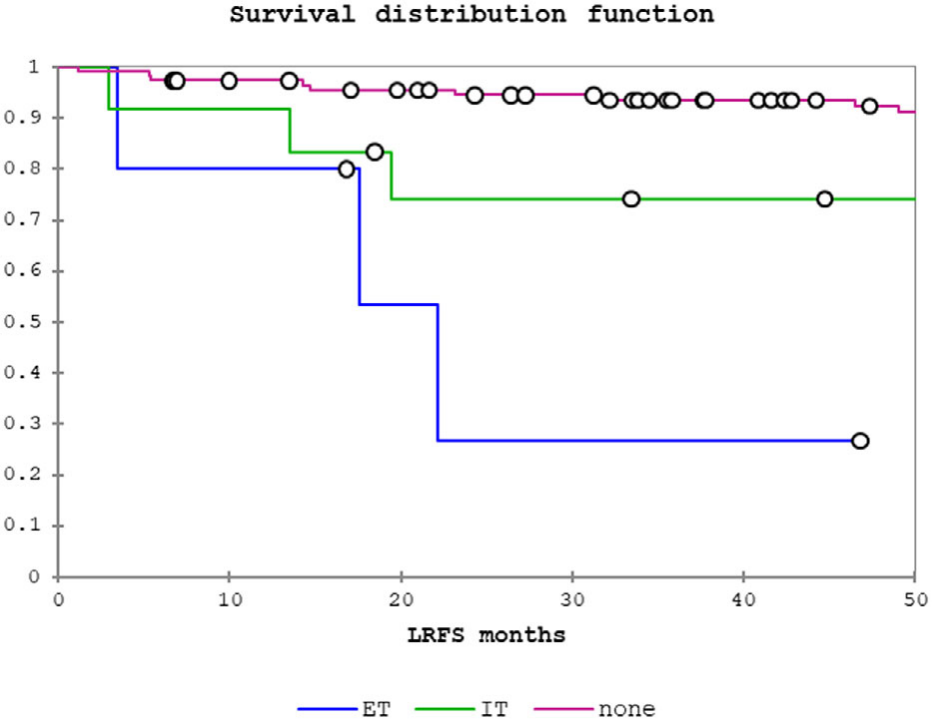
Locoregional recurrence‐free survival (LRS): no perineural invasion (PNI) versus intratumoural PNI versus extratumoural PNI.

**Figure 3:**
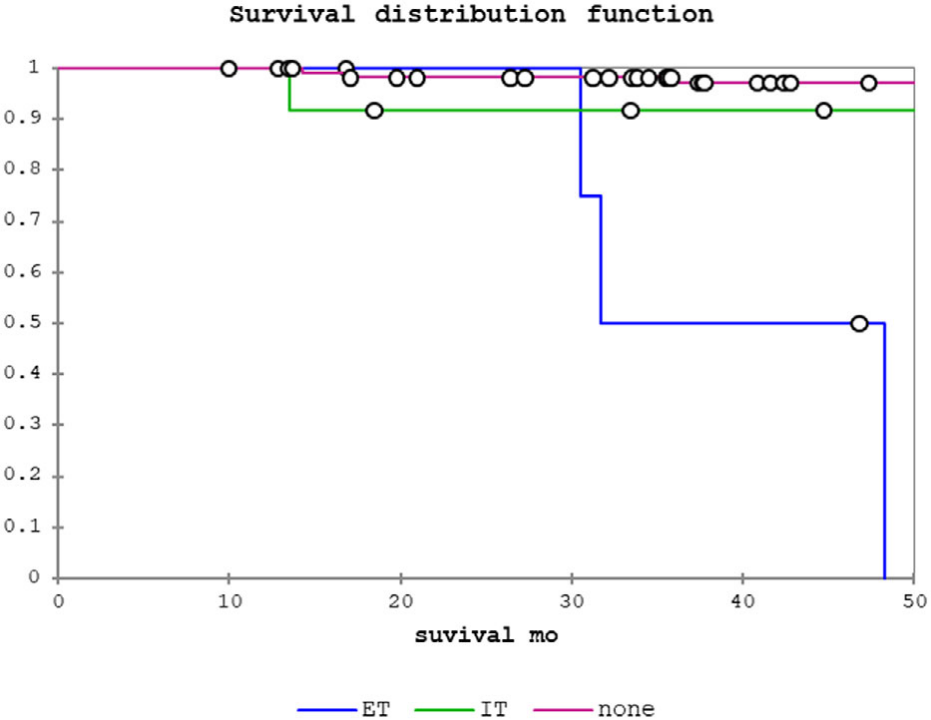
Disease‐specific survival (DSS): no perineural invasion (PNI) versus intratumoural PNI versus extratumoural PNI.

**Figure 4:**
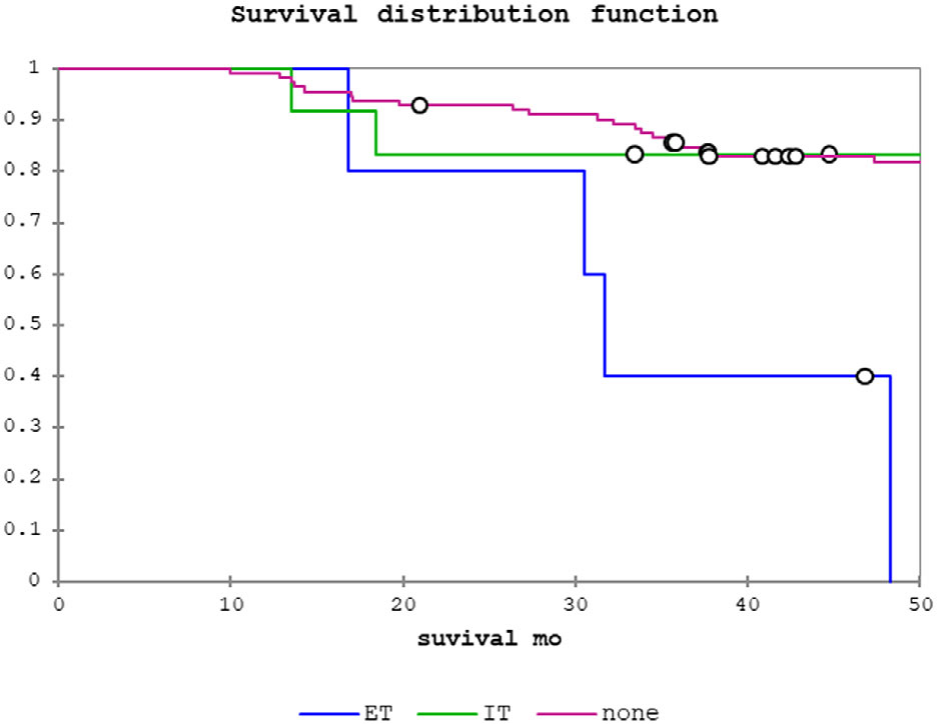
Overall survival (OS): no perineural invasion (PNI) versus intratumoural PNI versus extratumoural PNI.

Table 3 shows results of the univariate analysis of clinicopathological factors studied and associations with LRS, disease‐specific survival (DSS) and overall survival (OS) for the pN0/Nx patient cohort. Any PNI was predictive of LRS (odds ratio (OR) = 5.50, 95% confidence interval (CI) = 2.29–13.24, *P =* 0.0001), DSS (OR = 7.22, 95% CI = 2.25–23.19, *P =* 0.001) and OS (OR = 2.48, 95% CI = 1.24–4.93, *P =* 0.01). Regarding PNI location, IT PNI was significantly associated with LRS (OR = 4.18, 95% CI = 1.50–11.63, *P =* 0.006) but not with DSS or OS. In contrast, ET PNI was significantly associated with LRS (OR = 13.10, 95% CI = 3.46–49.52, *P* = 0.0002), DSS (OR = 28.95, 95% CI = 6.07–138.06, *P =* < 0.0001) and OS (OR = 8.31, 95% CI = 2.82–24.54, *P =* 0.0001). Figures 2, 3, 4 show the Kaplan–Meier curves for LRS, DSS and OS, respectively, for no PNI, IT PNI and ET PNI.

**Table 3:** Univariate analysis of impact of pathological factors and adjuvant radiotherapy on LRS, DSS and OS, TNM8 T1/T2 N0/Nx cases

		LRS OR (95% CI)	*P*‐value	DSS OR (95% CI)	*P*‐value	OS OR (95% CI)	*P*‐value
Radiotherapy		1.33 (0.54, 3.25)	0.53	0.67 (0.15, 3.05)	0.60	0.60 (0.29, 1.22)	0.16
Non‐cohesive invasive front		1.40 (0.60, 3.27)	0.44	2.21 (0.70, 6.98)	0.18	1.16 (0.65, 2.05)	0.62
Depth > 5 mm		1.53 (0.68, 3.48)	0.31	1.54 (0.49, 4.89)	0.46	0.78 (0.46, 1.31)	0.34
CAP margin		1.25 (0.37, 4.24)	0.73	1.63 (0.35, 7.59)	0.53	0.87 (0.35, 2.20)	0.78
Any PNI		5.50 (2.29, 13.24)	0.0001	7.22 (2.25, 23.19)	0.001	2.48 (1.24, 4.93)	0.01
PNI location	None	Reference		Reference		Reference	
	IT	4.18 (1.50, 11.63)	0.006	3.67 (0.76, 17.79)	0.11	1.72 (0.73, 24.54)	0.21
	ET	13.10 (3.46, 49.52)	0.0002	28.95 (6.07, 138.06)	< 0.0001	8.31 (2.82, 24.54)	0.0001
Multifocal PNI (reference: no PNI and unifocal PNI)		4.28 (1.57, 11.65)	0.004	5.23 (1.40, 19.59)	0.01	2.57 (1.16, 5.70)	0.02
Focality of PNI	None	Reference		Reference		Reference	
	ET, UF+MF	13.16 (3.48, 49.83)	0.0001	29.12 (6.09, 139.29)	< 0.0001	8.30 (2.81, 24.51)	0.0001
	IT UF	4.89 (1.09, 21.93)	0.038	5.03 (0.61, 41.72)	0.14	1.83 (0.66, 5.10)	0.25
	IT MF	3.81 (1.10, 13.29)	0.036	2.90 (0.36, 23.64)	0.32	1.53 (0.37, 6.35)	0.34
No neck dissection		0.61 (0.25, 1.47)	0.27	0.88 (0.26, 2.94)	0.83	1.91 (1.14, 3.22)	0.02

PNI, perineural invasion; OR, odds ratio; CI, confidence interval; IT, intratumoural; ET, extratumoural; UF, unifocal; MF, multifocal; LRS, locoregional recurrence‐free survival; DSS, disease‐specific survival; OS, overall survival; CAP, College of American Pathologists (CAP).

Multifocal PNI (versus no PNI and unifocal PNI) was also significantly associated with all three outcome parameters evaluated: LRS (OR = 4.28, 95% CI = 1.57–11.65, *P* = 0.004), DSS (OR = 5.23, 95% CI = 1.40–19.59, *P* = 0.01) and OS (OR = 2.57, 95% CI = 1.16–5.70, *P* = 0.02). When IT PNI was analysed separately based on focality, both unifocal and multifocal IT PNI correlated with LRS with *P*‐values of 0.038 and 0.036, respectively, but were non‐significant for DSS and OS. There were too few cases of ET PNI to allow meaningful subanalysis in this group. Kaplan–Meier curves of LRS, DSS and OS according to extent of PNI are shown in the Supporting information, Figures S1–3.

The only remaining correlation of note was a significant association between the pNx cohort and OS (OS = 1.91, 95% CI = 1.41–3.22, *P* = 0.02). All other variables analysed, including radiotherapy, non‐cohesive invasive front, depth of invasion > 5 mm and margin positivity, were non‐significant.

On multivariate analysis of the pN0/Nx cohort, both IT PNI (OR = 5.69, 95% CI = 1.50–21.63, *P* = 0.01) and ET PNI (OR = 20.57, 95% CI = 3.48–121.73, *P* = 0.001) remained significant for LRS. For DSS and OS, only ET PNI was significant (DSS OR = 40.47, 95% CI = 5.17–316.96, *P* = 0.0004 and OS OR = 11.92, 95% CI = 2.18–65.22, *P* = 0.004). In contrast, multifocal PNI lost significance for all outcomes analysed. Correlation of no neck dissection (pNx subgroup) with OS was also maintained (OR = 2.38, 95% CI = 1.31–4.33, *P* = 0.01); see Table 4.

**Table 4:** Multivariate analysis of impact of pathological factors and adjuvant radiotherapy on LRS, DSS and OS, TNM8 T1/T2 N0/Nx cases

		LRS OR (95% CI)	*P*‐value	DSS OR (95% CI)	*P*‐value	OS OR (95% CI)	*P*‐value
Radiotherapy		1.34 (0.53, 3.42)	0.54	0.71 (0.15, 3.45)	0.67	0.81 (0.38, 1.74)	0.29
PNI	None	Reference		Reference		Reference	
	IT	5.69 (1.50, 21.63)	0.01	4.93 (0.80, 30.41)	0.09	2.38 (0.70, 8.18)	0.17
	ET	20.57 (3.48, 121.73)	0.001	40.47 (5.17, 316.96)	0.0004	11.92 (2.18, 65.22)	0.004
Multifocal PNI (reference: no PNI and unifocal PNI)		1.73 (0.38, 7.90)	0.48	0.63 (0.09, 4.22)	0.64	1.11 (0.26, 4.68)	0.89
No neck dissection						2.38 (1.31, 4.33)	0.01

PNI, perineural invasion; LRS, locoregional recurrence‐free survival; DSS, disease‐specific survival; IT, intratumoural; ET, extratumoural; OS, overall survival; CI, confidence interval; OR, odds ratio.

Analysis was repeated on the overall pT1/T2 pN0/Nx/N+ patient cohort. The results in relation to PNI were consistent with those demonstrated in the pN0/Nx cohort, but additionally IT PNI demonstrated significance for DSS and OS on univariate analysis and for DSS on multivariate analysis. Other factors that correlated with survival outcomes were nodal positivity and extranodal extension (ENE) (Supporting information, Tables S1 and S2).

## Discussion

Perineural invasion is well documented as an important prognostic variable in OCSCC and is associated with poor locoregional control and decreased DSS and OS.^1^, ^2^, ^3^, ^4^, ^5^, ^6^, ^7^, ^8^, ^9^, ^10^ It also guides adjuvant therapy decision‐making, with improved outcomes demonstrated in patients with PNI undergoing postoperative chemoradiation in some, but not all, studies.^24^, ^25^, ^26^, ^27^ In a meta‐analysis of OCSCC cases with PNI, which included 690 patients from nine studies, Festa *et al*. reported postoperative radiotherapy to be associated with better disease‐free survival (DFS), but was non‐significant for locoregional control, DSS and OS^26^. Holcomb *et al*. also demonstrated a significantly decreased hazard ratio for DFS among patients with PNI‐positive disease undergoing adjuvant radiation in an early‐stage OCSCC cohort.^27^

In the present study, we have also demonstrated a significant correlation between the presence of PNI and patient outcomes including LRS, DSS and OS. Similarly to Festa *et al*., we found radiotherapy to be non‐significant in our cohort of early‐stage OCSCC patients. However, our findings in relation to radiotherapy may be biased by the retrospective nature of the study.

The presence of PNI is a required data element in the CAP guidelines, but location of PNI, although currently suggested for reporting, remains non‐core. In contrast, the RCPath acknowledges that the literature is conflicting, but nonetheless recommend routine reporting of ET PNI. Evidence supportive of mandatory reporting of PNI location includes the studies of Miller *et al*. and Park *et al*. The former found a trend towards reduced DFS in association with ET PNI.^11^ In cases with this finding, the latter demonstrated worse DFS on multivariate analysis.^12^ Although the number of cases of ET PNI in our cohort was very small (five cases), the impact of ET PNI was highly significant. On multivariate analysis, we found PNI that was ET in location to correlate significantly with LRS, DSS and OS. IT PNI showed significance on multivariate analysis for LRS only and, of note, was non‐significant for DSS and OS.

PNI extent is not a core data element for reporting in the CAP or RCPath guidelines at this time. However, there are accumulating data that the extent of PNI is prognostic. Park *et al*. found multifocal PNI to be associated with worse DFS and DSS on multivariate analysis. The correlation with worse DFS was maintained irrespective of whether or not adjuvant treatment was given.^12^ Aivazian *et al*. also demonstrated a significant association between multifocal PNI and both local failure and DSS independently of postoperative radiation.^13^ Wei *et al*. quantified PNI when present as low (one to five foci) or high (more than five foci) in a T1–T2 OCSCC cohort. The PNI focus number predicted for cervical nodal metastases, DSS and OS on multivariable assessment.^14^ Hasmat *et al*., in a cohort of 993 OCSCC patients, including high stage disease, found multifocal PNI to predict for both DSS and OS.^15^ In contrast, Arun *et al*. did not find histological subclassification of PNI, including location or focality, to be prognostically significant in a retrospective study of 207 OCSCC patients.^28^ In our cohort we found multifocal PNI to correlate significantly with all three outcome parameters evaluated on univariate assessment. However, significance was lost on multivariate analysis, which could be due at least in part to an association between multifocality and ET location of PNI. When IT PNI was further assessed based on extent, both unifocal and multifocal IT PNI versus no PNI correlated with LRS, but with similar ORs, suggesting that focality may be less important than location. Finally, there were too few cases of ET PNI to attempt meaningful analysis of focality within this group. Our results in relation to PNI were consistent between the overall cohort, which included node‐positive cases and the pN0/pNx subgroup, but with additional correlations between IT PNI and survival outcomes for the former.

In our study, an unexpected finding was a significant association between those patients who had not undergone neck dissection (pNx cases) and OS on both univariate and multivariate analysis. We postulate that this may be explained by a substantial proportion of the pNx cohort not having been offered neck dissection due to poor performance status. This theory is supported by the finding that this subgroup did not have higher rates of locoregional recurrence and DSS was not a significant correlate.

The main limitations of this study are its retrospective nature and the relatively small number of PNI‐positive patients within the location and focality subcategories. However, the presence of ET PNI was found to be highly significant for all outcome parameters evaluated on multivariable analysis, despite the very small number of cases. We acknowledge that we were probably underpowered in our assessment of the impact of multifocal PNI, and therefore cannot draw any firm conclusions regarding its potential significance. That not all cases underwent slide review may be regarded as an additional limitation. However, this would not effectively mitigate intra‐ and interobserver variability in the evaluation of PNI.^19^, ^20^

## Conclusion

PNI is associated with worse survival outcomes in early‐stage OCSCC. Our results additionally highlight the value of subclassifying PNI by location, with ET PNI representing the major adverse prognosticator in our cohort. IT PNI was also prognostic, but not for overall survival. The association between PNI extent and patient outcomes is less clear‐cut. In conclusion, our findings support the histological reporting of PNI location (IT versus ET) as a mandatory data element, whereas the impact of PNI extent (unifocal versus multifocal) requires further study. Given its prognostic impact, identification of ET PNI in early‐stage OCSCC should trigger consideration for adjuvant therapy.

## Supporting information


**Supporting information, Figure 1.** LRS: No PNI versus unifocal IT PNI versus multifocal IT PNI versus ET PNI.
**Supporting information, Figure 2.** DSS: No PNI versus unifocal IT PNI versus multifocal IT PNI versus ET PNI.
**Supporting information, Figure 3.** OS: No PNI versus unifocal IT PNI versus multifocal IT PNI versus ET PNI.

## Data Availability

The data that support the findings of this study are available on request from the corresponding author. The data are not publicly available due to privacy or ethical restrictions.
