# Supplementary material for: Significance of location and extent of perineural invasion in early‐stage oral cavity squamous cell carcinoma
Source: Histopathology. 2025 Jan 6;86(6):993–1000. doi: 10.1111/his.15406 (PMC11964579; doi:10.1111/his.15406)
Supplement: Supplementary file 1 — Supporting information, Figure 1. LRS: No PNI versus unifocal IT PNI versus multifocal IT PNI versus ET PNI. Supporting information, Figure 2. DSS: No PNI versus unifocal IT PNI versus multifocal IT PNI versus ET PNI. Supporting information, Figure 3. OS: No PNI versus unifocal IT PNI versus multifocal IT PNI versus ET PNI. [file HIS-86-993-s001.docx]

Supplementary table 1: Univariate analysis of impact of pathological factors and adjuvant radiotherapy on (LRS), (DSS), and (OS), TNM8 T1/T2 N0/N+ cases

|  | LRS OR (95% CI) | p-value | DSS OR (95% CI) | p-value | OS OR (95% CI) | p-value |
| --- | --- | --- | --- | --- | --- | --- |
| Radiotherapy | 1.76 (0.89, 3.48) | 0.10 | 1.97 (0.85, 4.57) | 0.11 | 0.99 (0.61, 1.60) | 0.96 |
| Non-cohesive invasive front | 1.51 (0.77, 2.98) | 0.23 | 2.43 (1.05, 5.64) | 0.04 | 1.25 (0.79, 1.98) | 0.34 |
| Depth > 5mm | 1.51 (0.76, 3.01) | 0.24 | 1.91 (0.80, 4.57) | 0.14 | 0.93 (0.60, 1.46) | 0.78 |
| CAP margin | 1.37 (0.48, 3.94) | 0.56 | 1.59 (0.47, 5.42) | 0.46 | 0.90 (0.41, 1.96) | 0.79 |
| Any PNI | 6.10 (3.00, 12.37) | <0.0001 | 7.84 (3.33, 18.43) | <0.0001 | 2.40 (1.42, 4.06) | 0.001 |
| Intratumoral PNI *(vs. no PNI)* | 5.58 (2.64, 11.78) | <0.0001 | 6.97 (2.78, 17.34) | <0.0001 | 2.16 (1.22, 3.83) | 0.008 |
| Extratumoral PNI *(vs. no PNI)* | 10.41 (2.93, 37.05) | 0.0003 | 13.34 (3.50, 50.81) | 0.0001 | 4.35 (1.54, 12.34) | 0.006 |
| Multifocal PNI | 4.54 (2.13, 9.68) | <0.0001 | 7.09 (2.99, 16.79) | <0.0001 | 2.59 (1.44, 4.64) | 0.001 |
| pN+ | 2.61 (1.23, 5.52) | 0.01 | 5.61 (2.40, 13.11) | <0.0001 | 2.41 (1.44, 4.02) | 0.001 |
| ECS | 5.27 (1.75, 15.83) | 0.003 | 15.67 (5.16, 47.54) | <0.0001 | 11.46 (5.27, 24.90) | <0.0001 |

Supplementary table 2: Multivariate analysis of impact of pathological factors and adjuvant radiotherapy on (LRS), (DSS), and (OS), TNM8 T1/T2 N0/N+ cases

|  |  | LRS OR (95% CI) | p-value | DSS OR (95% CI) | p-value | OS OR (95% CI) | p-value |
| --- | --- | --- | --- | --- | --- | --- | --- |
| Radiotherapy |  | 1.20 (0.52, 2.79) | 0.67 | 0.82 (0.27, 2.53) | 0.12 | 0.64 (0.37, 1.13) | 0.13 |
| Non-cohesive invasive front |  |  |  | 0.59 (0.20, 1.77) | 0.89 |  |  |
| PNI | None | reference |  | reference |  | reference |  |
|  | Intra-tumoural | 6.19 (2.14, 17.91) | 0.001 | 4.73 (1.17, 19.09) | 0.03 | 1.66 (0.64, 4.29) | 0.30 |
|  | Extra-tumoural | 19.87 (4.01, 98.42) | 0.0002 | 33.36 (4.72, 235.56) | 0.0004 | 7.06 (1.77, 28.10) | 0.006 |
| Multifocal PNI (reference: no PNI and unifocal PNI) |  | 0.54 (0.16, 1.80) | 0.31 | 0.76 (0.17, 3.47) | 0.73 | 0.69 (0.22, 2.12) | 0.51 |
| pN+ |  | 1.46 (0.49, 4.30) | 0.50 | 5.05 (1.27, 20.14) | 0.02 | 1.92 (0.94, 3.94) | 0.07 |
| ECS |  | 2.29 (0.53, 9.83) | 0.27 | 4.19 (0.88, 20.08) | 0.07 | 8.63 (2.89, 25.81) | 0.0001 |

Supplementary figure 1: LRS: No PNI versus unifocal IT PNI versus multifocal IT PNI versus ET PNI


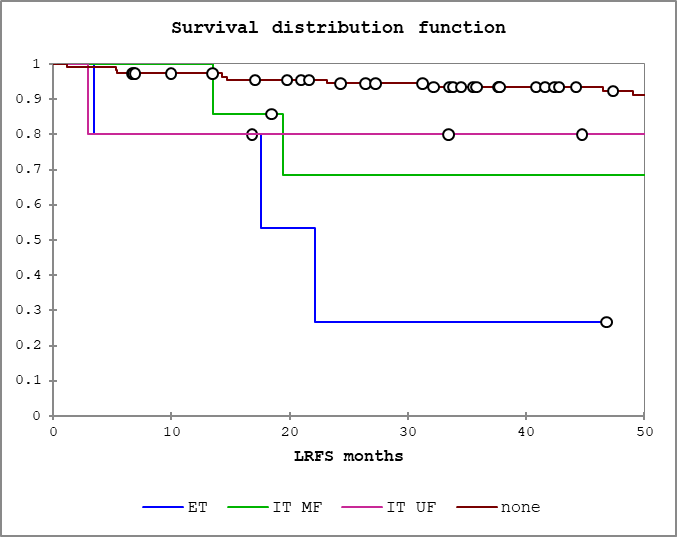


Supplementary figure 2: DSS: No PNI versus unifocal IT PNI versus multifocal IT PNI versus ET PNI


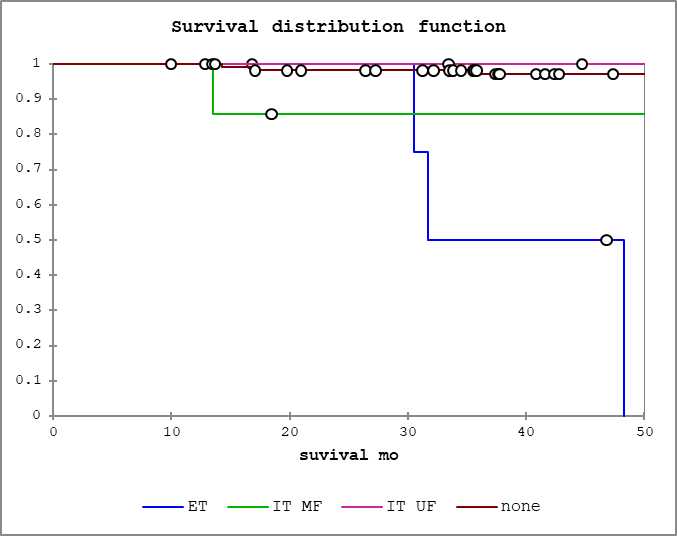


Supplementary figure 3: OS: No PNI versus unifocal IT PNI versus multifocal IT PNI versus ET PNI


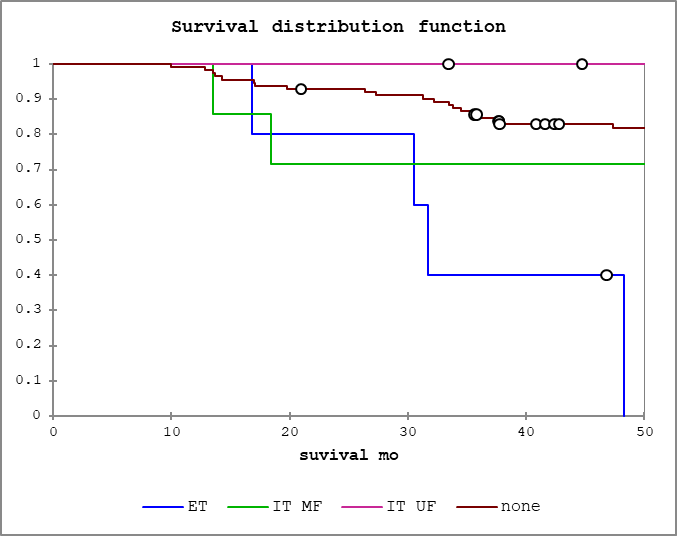


Figure legends:

Supplementary figure 1: LRS: No PNI versus unifocal IT PNI versus multifocal IT PNI versus ET PNI

Supplementary figure 2: DSS: No PNI versus unifocal IT PNI versus multifocal IT PNI versus ET PNI

Supplementary figure 3: OS: No PNI versus unifocal IT PNI versus multifocal IT PNI versus ET PNI
